# Multiomics data integration to reveal chromatin remodeling and reorganization induced by gene mutational synergy

**DOI:** 10.1016/j.xpro.2022.101770

**Published:** 2022-10-14

**Authors:** Haiyang Yun, Shabana Vohra, David Lara-Astiaso, Brian J.P. Huntly

**Affiliations:** 1 Department of Medicine V, Hematology, Oncology and Rheumatology, University Hospital Heidelberg, Heidelberg, Germany; 2 Molecular Medicine Partnership Unit, European Molecular Biology Laboratory, Heidelberg, Germany; 3 Department of Haematology, University of Cambridge, Cambridge, UK; 4 Wellcome - MRC Cambridge Stem Cell Institute, Cambridge, UK

**Keywords:** Bioinformatics, Cancer, Genomics

## Abstract

Recurrent gene mutations often cooperate in a predefined stepwise and synergistic manner to alter global transcription, through directly or indirectly remodeling epigenetic landscape on linear and three-dimensional (3D) scales. Here, we present a multiomics data integration approach to investigate the impact of gene mutational synergy on transcription, chromatin states, and 3D chromatin organization in a murine leukemia model. This protocol provides an executable framework to study epigenetic remodeling induced by cooperating gene mutations and to identify the critical regulatory network involved.

For complete details on the use and execution of this protocol, please refer to Yun et al. (2021).

## Highlights

• A computational framework for studying chromatin remodeling by mutational synergy
• Pipelines for linking mutation-associated chromatin elements with target genes
• Method for identification of critical network nodes involving chromatin remodeling

## Abstract (editor highlights)

Publisher’s note: Undertaking any experimental protocol requires adherence to local institutional guidelines for laboratory safety and ethics.

## Graphical abstract

**Figure:**
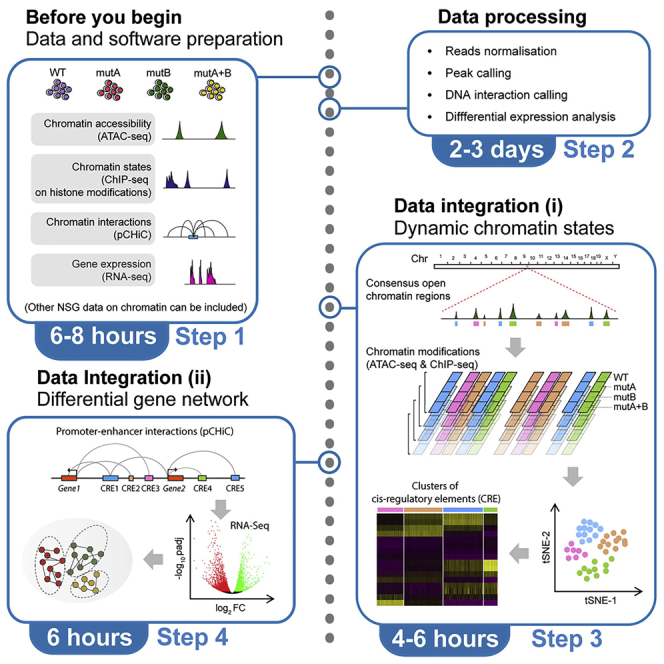

## Before you begin

The protocol below describes the specific steps for performing the integrative analysis of chromatin accessibility, chromatin states, DNA looping and transcriptome across four cellular states in a murine allelic series that models the two most common mutations in acute myeloid leukemia (AML): *Flt3-ITD* and *Npm1c,* both of which are present in 15%–20% of all AML cases. Either of the two mutations alone caused mild and pre-malignant phenotype, whereas in combination they demonstrated strong synergistic effect to induce aggressive AML. The hematopoietic stem and progenitor cells (HSPCs), a bulk cell population represented by the lineage negative (Lin-) fraction of bone marrow cells harvested from wildtype (WT), single mutant (*Flt3-ITD* or *Npm1c*), and double mutant (DM, both *Flt3-ITD* and *Npm1c*) mice, were used to generate all the multiomics data as an exemplar in this protocol.

This section includes minimum requirements for computer hardware, pre-installation of software (including tools and algorithms) for data processing, as well as a collection of exemplar next generation sequencing (NGS) data to be analyzed.

### Computer system

Exemplar data analysis in the protocol is performed in a computational environment with system specifications indicated in the key resources table.

***Note:*** We recommend a computer system containing a minimum configuration of 16 GB local memory and 12 CPU cores.

### Software

Computational tools and software for raw data processing and further integrative analysis are listed in the key resources table.

***Note:*** To recapitulate the procedures undertaken in our published work (Yun et al., 2021) and to confirm the data reproducibility, the same software versions are installed when possible, otherwise the latest version are utilized. In addition, we have tested all the R scripts with the latest R version (v.4.1.3), and we can seamlessly reproduce the results. And this also applies to the latest versions of R packages including CHiCAGO (v.1.24.0), Seurat (v.4.1.1), DEseq2 (v.1.36.0), and DiffBind (v3.6.3). As other software is mainly used for the initial processing of NGS reads, such as mapping, initial QC analysis and data filtering, we would anticipate that their newer versions will retain the main analytical and statistical power with the same key parameters, though not yet been tested on our exemplar data. Nevertheless, we cannot guarantee that there will be no compatibility issues when running the latest versions of some software or algorithms.

### Algorithms and scripts

Algorithms and scripts required in the protocol are available in GitHub (https://github.com/haiyang-yun/3D_chromatin_in_AML) and are indicated in the key resources table.

### Data collection

**Timing: 6–8 h**

Collect the bulk NGS data of each genomic profiling approach below in four cellular states (HSPCs from WT, *Flt3-ITD, Npm1c,* and DM mice) for processing. All the experiments were performed as described (Yun et al., 2021). All NGS data in the protocol were generated on Hiseq 2500 or 4000 platforms, with raw reads available in *.FASTQ* files with two biological replicates for each assay condition for each genotype. Download the relevant data from data repositories as indicated in the key resources table.

1. Chromatin accessibility profiled by assay for transposon accessible chromatin (ATAC-seq).
2. Multiple chromatin activation states detected by chromatin immunoprecipitation and mass parallel sequencing (ChIP-seq) on histone H3 lysine 4 mono- or trimethylation (H3K4me1 or H3K4me3) and histone H3 lysine 27 acetylation (H3K27ac).
3. Promoter-anchored 3D chromatin interaction detected by Promoter capture HiC (pCHiC).
4. Global gene expression profiled by RNA high-throughput sequencing (RNA-seq).

## Key resources table

**Table:** 

REAGENT or RESOURCE	SOURCE	IDENTIFIER
**Software and algorithms**		

FastQC	Andrews (2010)	http://www.bioinformatics.babraham.ac.uk/projects/fastqc
Bowtie2 (v.2.1.0)	(Langmead and Salzberg, 2012)	http://bowtie-bio.sourceforge.net/bowtie2/index.shtml
Picard tools (v.2.2.1)	Picard Toolkit, 2019	https://broadinstitute.github.io/picard
MACS2 (v.2.0.1)	(Zhang et al., 2008)	https://pypi.org/project/MACS2
STAR (v.2.4.0)	(Dobin et al., 2013)	https://github.com/alexdobin/STAR
DESeq2 (v.1.12.4)	(Love et al., 2014)	https://bioconductor.org/packages/release/bioc/html/DESeq2.html
Python (v.3.9.5)	Van Rossum and Drake (2009)	https://www.python.org/
HTSeq (v.0.6.0)	(Anders et al., 2015)	https://htseq.readthedocs.io/en/master/
R (v.3.6.1)	R Core Team (2019)	https://www.R-project.org
HiCUP (v.0.5.8)	(Wingett et al., 2015)	https://www.bioinformatics.babraham.ac.uk/projects/hicup
CHiCAGO (v.1.1.1)	(Cairns et al., 2016)	https://functionalgenecontrol.group/chicago
Seurat (v.3.2.3)	(Satija et al., 2015)	https://satijalab.org/seurat/articles/get_started.html
DiffBind (v.2.0.1)	(Ross-Innes et al., 2012)	https://bioconductor.org/packages/release/bioc/html/DiffBind.html
featureCounts (Subread package v.2.0.1)	(Liao et al., 2014)	http://subread.sourceforge.net/featureCounts.html
ShinyGO v0.76	(Ge et al., 2020)	http://bioinformatics.sdstate.edu/go/
get_data.sh	(Yun et al., 2021)	https://github.com/haiyang-yun/3D_chromatin_in_AML/tree/main/ChIP-seq
process_aligned_reads.sh	(Yun et al., 2021)	https://github.com/haiyang-yun/3D_chromatin_in_AML/tree/main/ChIP-seq
runRNA_STAR_paired.pl	(Yun et al., 2021)	https://github.com/haiyang-yun/3D_chromatin_in_AML/tree/main/RNA-seq
RNAseq_differential_analysis.R	(Yun et al., 2021)	https://github.com/haiyang-yun/3D_chromatin_in_AML/tree/main/Differential_analysis/
Digest.mm10.rmap	(Yun et al., 2021)	https://github.com/haiyang-yun/3D_chromatin_in_AML/tree/main/Other
CHiC.mm10.baitmap	(Yun et al., 2021)	https://github.com/haiyang-yun/3D_chromatin_in_AML/tree/main/Other
ATAC_consensus_peakmax.R	(Yun et al., 2021)	https://github.com/haiyang-yun/3D_chromatin_in_AML/tree/main/Other
ATAC_peaksummit_to_saf.R	(Yun et al., 2021)	https://github.com/haiyang-yun/3D_chromatin_in_AML/tree/main/Other
Multiomics_Seurat_analysis_v2022.R	(Yun et al., 2021)	https://github.com/haiyang-yun/3D_chromatin_in_AML/tree/main/Other
Cluster_CREs_genes_diffexp.R	(Yun et al., 2021)	https://github.com/haiyang-yun/3D_chromatin_in_AML/tree/main/Other
ATAC_consensus_summit2kb_adj_cpm_merge_transpose.txt	(Yun et al., 2021)	https://github.com/haiyang-yun/3D_chromatin_in_AML/tree/main/Other
pCHiC_fragID_Gene.txt	(Yun et al., 2021)	https://github.com/haiyang-yun/3D_chromatin_in_AML/tree/main

**Deposited data**		

Raw ATAC-seq data	(Yun et al., 2021)	GSE146616
Raw ChIP-seq data	(Yun et al., 2021)	GSE146663
Raw pCHiC data	(Yun et al., 2021)	GSE146662
Raw RNA-seq data	(Yun et al., 2021)	GSE146668

**Other**		

Intel(R) Xeon(R) Silver 4214 CPU @ 2.20 GHz (48 CPUs × 2 Threads), 756 GB memory, Debian GNU/Linux 10 (buster)	N/A	N/A

## Step-by-step method details

Herein we describe step-by-step analytical procedures starting from raw data processing all the way through to integrated data analysis. The raw data undergo serial processing steps covering: quality control, read mapping, data filtering, normalization, and statistical calling. Subsequently, the processed data are first subjected to integrated analysis on dynamic chromatin states, to reveal differential clusters of cis-regulatory elements (CREs) that demonstrate similar dynamic chromatin modifications. Afterwards, the specific clusters of CREs with characteristic gain or loss of enhancer signatures are annotated to target genes, using either linear or spatial proximity information. Differential mRNA expression is further analyzed for these genes along with their associated functional network. The relevant biological information of the data used and their functional interpretation are discussed in great detail in (Yun et al., 2021).

### Data processing

**Timing: 2–3 days**

In this section, the raw data from different genomic approaches are processed in a stepwise manner and are transformed into a format compatible with the subsequent integrative analysis. In brief, a QC step is applied to check the ChIP-seq and ATAC-seq data quality prior to reads mapping to mouse genome, followed by the removal of duplicated reads. Subsequently, genotype-specific open chromatin states are identified by calling significant peaks on ATAC-seq in each cellular condition. Next, transcriptome data profiled by RNA-seq are processed in a similar fashion but with different tools. In addition, the RNA-seq read counts are extracted for all annotated genes and differential expression of protein-coding genes between single or double mutant cells and wildtype cells is analyzed. Finally, chromatin interaction data stored in raw .*FASTQ* files of pCHiC are converted into readable promoter-associated DNA interaction files. The data processing steps are described in great detail below.

1. Process raw reads in ATAC-seq and ChIP-seq data for each genotype.
  a. Perform QC and reads mapping by running custom scripts (“get_data.sh”) on the input *.FASTQ* files.
    > get_data.sh -g [GENOTYPE] -m [OUTPUT_FOLDER] -i [INPUT_FASTQ] -x mm10
    ***Note:*** QC analysis is carried out with FastQC package, and raw reads are mapped to *Mus musculus* (house mouse) genome assembly GRCm38 (mm10) using Bowtie2, with parameters allowing to keep reads for at most 2 alignment and 1 mismatch in the seed (20 bp default).
  b. Filter the mapped reads by removing duplicate reads with custom scripts (“process_aligned_reads.sh”) as below.
    > process_aligned_reads.sh -g [GENOTYPE] -m [OUTPUT_FOLDER] -x mm10
    ***Note:*** This process utilizes Picard tools with the “MarkDuplicates” function for data filtering, and generates sorted .*BAM* files.
  c. Identify significant ATAC-seq peaks by running MACS2 callpeak on filtered *.BAM* files with a pre-defined *p* value at 1e-20.
    > macs2 callpeak -t [INPUT_BAM] -g mm -f BAM -n [OUTPUT_FILE_NAME] -p 1e-20 --nomodel --nolambda --bdg
    ***Note:*** The parameter --nomodel here is specified for single-read ATAC-seq data (the exemplar data), without modeling the fragment size and by default extends the reads for 200 bp. This may not accurately reflect the actual length of nucleosome-free regions.
2. Process RNA-seq raw data and analyze differential expression of protein-coding genes between mutant and wildtype samples.
  a. Process RNA-seq data by running custom scripts (“runRNA_STAR_paired.pl”) on paired *.FASTQ* files (r_1 and r_2) for each genotype.
    ***Note:*** This process covers QC analysis using FastQC, then reads mapping and uniquely mappable reads extraction using STAR package which allows at most 2 mismatches, and subsequently read counts computation for all annotated genes using a python package HTSeq.
    > runRNA_STAR_paired.pl [INPUT_r_1_FASTQ] [INPUT_r_2_FASTQ] [GENOTYPE] mm10 STAR-GENOMES-mm10.gencode.vM7.comprehensive gencode.vM7.comprehensive.annotation.gtf [exons y/n]
  b. Analyze pairwise differential gene expression between any mutant condition (*Npm1c*, *Flt3-ITD*, or DM) and WT counterpart by running custom scripts (“RNAseq_differential_analysis.R”) on *.HTSEQ.COUNTS* files generated in step 2a.
    > Rscript RNAseq_differential_analysis.R
    ***Note:*** Bioconductor package DESeq2 is the core analytical tool utilized in this step. The output files are in *.CSV* format (e.g., “WT.DM.PC.diffExp.csv”).
3. Process the promoter-associated chromatin interaction data profiled by pCHiC assays in each cellular condition.
  a. Process pCHiC raw data (paired reads, r_1 and r_2) using HiCUP pipeline to map and filter the data and eventually output valid HiC fragments (termed di-tags) stored in *.BAM* files.
    > hicup_digester --genome Mouse_GRCm38 --re1 AˆAGCTT,HindIII [mm10_GENOME.fa]
    > hicup --bowtie2 [BOWTIE2_PATH] --index [mm10_REFERENCE_GENOME_PATH] --digest [mm10_HINDIII_DIGESTION_FILE] --format Sanger --longest 800 --shortest 150 [INPUT_r_1_FASTQ] [INPUT_r_2_FASTQ]
    ***Note:*** The format of all input files is described in the HiCUP pipeline documentation (https://www.bioinformatics.babraham.ac.uk/projects/hicup/read_the_docs/html/index.html). To execute HiCUP, the input HindIII_digestion_file needs to be generated using hicup_digester (included in the hicup software) using the first code above.
  b. Transform valid HiC di-tags into statistically significant chromatin interactions associated with all mouse promoters using Bioconductor package CHiCAGO.
    i. Convert filtered read pairs in .*BAM* files generated by HiCUP into the CHiCAGO input data format, *.CHINPUT.*
      > bam2chicago.sh [INPUT_BAM] CHiC.mm10.baitmap Digest.mm10.rmap [OUTPUT_FILE] nodelete
      ***Note:*** The availability of the shell script, as well as the description and preparation of input files can be referred to CHiCAGO online instruction (https://bitbucket.org/chicagoTeam/chicago/src/master/chicagoTools/). The rmap file (*.RMAP*) and baitmap file (*.BAITMAP*) are tab-separated files describing the restriction digestion fragments and the coordinates of the baited/captured restriction fragments, respectively, all with numeric IDs. Both files can be generated by a CHiCAGO script (“create_baitmap_rmap.pl”) which is accessible via clicking the link above.
    ii. Further statistical analysis is performed on *.CHINPUT* files from genotype replicates to generate a list of significant promoter-associated DNA interactions.
      > Rscript runChicago.R --design-dir [DESIGN_FILES_PATH] [CHINPUT_FILE_1, CHINPUT_FILE_2,…] [OUTPUT_FILE] WT.CHiC.R1.chinput,WT.CHiC.R2.chinput WT.CHiC.R1-2
      ***Note:*** Significant interactions are called when CHiCAGO scores are ≥5. The format of CHiCAGO input files is described in the CHiCAGO pipeline documentation (https://bitbucket.org/chicagoTeam/chicago/src/master/chicagoTools/).
      **CRITICAL:** Data processing by HiCUP and CHiCAGO are heavy computation tasks which favor usage of multiple CPU cores and large memory. The running time can be reduced to a reasonable duration in a computational environment with at least 24 threads and 48 GB RAM.

### Data integration (i)—Dynamic chromatin states

**Timing: 4–6 h**

We first apply a multilayered approach (Ma et al., 2020) to integrate the multiomics chromatin analysis at all cis-regulatory elements (CREs) in wildtype and mutant HSPCs. As CREs are usually rendered accessible by chromatin binding factors such as transcription factors (TFs), their presence can be implied by open chromatin sites, which are profiled by ATAC-seq. We therefore identify all open chromatin sites across four cellular conditions by creating a compendium of ATAC-seq consensus peak sets. Afterwards, the read counts for each chromatin condition (H3K4me1, H3K4me3, H3K27ac and ATAC-seq) of each genotype (WT, *Npm1c*, *Flt3-ITD* and DM) at these potential CREs are computed to build a data matrix for further clustering analysis. Subsequently, the data matrix is processed in a similar way as for single-cell RNA-sequencing with the Seurat package, treating all CREs (as columns, equivalent to cells in a typical Seurat workflow) as separate data points across all 16 assay conditions (as rows, 4 chromatin profiles × 4 phenotypes, equivalent to genes in Seurat). This allows dimensionality reduction to classify and visualize clusters of CREs with similar patterns across wildtype and mutant cells. Meanwhile, specific clusters of chromatin regions showing leukemia-specific alterations of chromatin activation marks are identified for downstream gene network analysis.

4. Create a catalog of ATAC-seq consensus peak sets across four cellular states and convert it into a data table listing 2-kilo base (kb) bins at these consensus peaks (±1 kb from peak summit) in a format of *.SAF* required for read counts extraction using featureCounts.
  a. Make a sample list (“samplesheet_ATAC.csv”) indicating which ATAC-seq samples to be processed and the path to the storage of filtered reads (in *.BAM* files) and peak files (created by MACS2), using the layout below (row 3–6 are examples).

**Table:** 

SampleID	Condition	Replicate	bamReads	ControlID	bamControl	Peaks
[*SAMPLE*]	[*GENOTYPE*]	1	[*BAM_FILE*]	NA	NA	[*PEAK_FILE*]
WT.ATAC.R1	WT	1	[path_to_bam_file]	NA	NA	[path_to_MACSpeak_file]
WT.ATAC.R2	WT	2	[path_to_bam_file]	NA	NA	[path_to_MACSpeak_file]
NPM1.ATAC.R1	NPM1	1	[path_to_bam_file]	NA	NA	[path_to_MACSpeak_file]
NPM1.ATAC.R2	NPM1	2	[path_to_bam_file]	NA	NA	[path_to_MACSpeak_file]
  b. By running custom scripts (“ATAC_consensus_peakmax.R”) on the sample list (“samplesheet_ATAC.csv”) generated in step 4a, a list of consensus peak sets (“ATAC_consensus_peaks.bed”) is computed on ATAC-seq peaks from all genotypes including all their replicates. Then supplement this list with the information of which sample has maximal ATAC-seq signal at each peak (“ATAC_consensus_peakmax.bed”).
    > Rscript ATAC_consensus_peakmax.R
    ***Note:*** This step is performed by running DiffBind within our custom scripts.
  c. Identify the peak summit of each consensus peak sets (summit of the sample with maximal ATAC-seq signal identified in step 4b) and convert this information to featureCounts input file (“ATAC_consensus_summit2kb_adj.saf”) by creating genome coordinates of 2-kb bins surrounding ATAC-seq consensus peak summits (±1 kb from peak summit), with the help of running custom scripts (“ATAC_peaksummit_to_saf.R”).
    > awk '{print $1"\t"$2"\t"$3"\t"$5"\t""[SAMPLE]"}' [PEAK_FILE] > [SAMPLE_PEAK_SUMMIT_BED]
    > cat [ALL_PEAK_SUMMIT_BED] | sort -k1,1 -k2,2n > ATAC_all_summit.bed
    > bedtools intersect -a ATAC_consensus_peakmax.bed -b ATAC_all_summit.bed -wa -wb > ATAC_consensus_peakmax_intersect_summit.bed
    > sort -k4,4 -k9,9rn ATAC_consensus_peakmax_intersect_summit.bed | sort -uk4,4 | awk '{print $6"\t"$7"\t"$8"\t"$4}' | sort -k1,1 -k2,2n > ATAC_consensus_peak_summit.bed
    # “ATAC_consensus_peak_summit.bed” is the input file for subsequent conversion to .*SAF*file
    > Rscript ATAC_peaksummit_to_saf.R
5. Extract the read counts for each genomic approach (H3K4me1, H3K4me3, H3K27ac, and ATAC-seq) in each cellular condition at the 2-kb bins of ATAC-seq consensus peaks from the corresponding *.BAM* files (with replicates merged and normalized as count per million total read counts) using featureCounts.

> featureCounts -a ATAC_consensus_summit2kb_adj.saf -F SAF -t exon -g GeneID --largestOverlap -o ATAC_consensus_summit2kb_adj_counts.txt [ALL_BAM_FILES]

6. Perform integrative analysis on multilayered chromatin profiling data of all four genotype samples by running custom scripts (“Multiomics_Seurat_analysis_v2022.R”) to identify clusters of CREs (accessible chromatin regions) with similar dynamic chromatin states across WT and mutant conditions.

> Rscript Multiomics_Seurat_analysis_v2022.R

# input files “ATAC_consensus_summit2kb_adj_counts.txt” and “ATAC_consensus_summit2kb_adj_counts.txt.summary” were generated in step 5 by featureCounts

***Note:*** A prerequisite to dimensionality reduction analysis is a data matrix containing CREs as column (equivalent to cells in a typical Seurat workflow) and samples as rows (equivalent to genes in Seurat), filling with normalized read counts (CPM) on merged replicates of each condition, in a layout format as listed below. An exemplar data matrix (“ATAC_consensus_summit2kb_adj_cpm_merge_transpose.txt”) is provided in the key resources table.

**Table:** 

	CRE_1	CRE_2	CRE_3	…	…	CRE_N
[*SAMPLE*]	[*CPM*]	[*CPM*]	[*CPM*]	[*CPM*]	[*CPM*]	[*CPM*]
WT.H3K4me1						
NPM1.H3K4me1						
…						
WT.ATAC						
NPM1.ATAC						
…

***Note:*** By analyzing our exemplar data, this step generates three plots as shown in Figure 1. Using a heuristic method (ElbowPlot() function in Seurat package), we observe an ‘elbow’ around PC7-8 (Figure 1A), suggesting that the majority of true signal is captured in the first 8 PCs. Subsequent analysis using FindClusters() function outputs 10 communities, followed by computation of 10 clusters by non-linear dimensionality reduction algorithms: UMAP or tSNE (Figure 1B). And we found individual clusters were more well separated in tSNE plot than in UMAP. Therefore, the 10 tSNE-clusters are further subjected to heatmap plotting, to demonstrate individually dynamic patterns across WT and mutant conditions (Figure 1C). Furthermore, we extract the genomic coordinates of Cluster-6 CREs as exemplar data to analyze their associated gene network. This creates a bed file (“Multiomics_Cluster-6_summit200bp.bed”) which contains genome coordinates of a 200 bp region surrounding ATAC-peak summit of Cluster-6 for downstream annotation analysis.

***Note:*** To link a set of CREs (tSNE clusters) with mutation condition, by qualitatively analyzing the dynamic pattern of chromatin profiles associated with mutation alone or in combination in the heatmap (Figure 1C), we identified several clusters which demonstrate synergistic impact of mutations on chromatin modulation. For example, we identified CREs showing gains of enhancer marks and accessibility by mutations (e.g., Flt3-ITD and DM), which were separated by marked gain of accessibility (Cluster-5) and H3K27ac (Cluster-6). In comparison, Cluster-8 and Cluster-1 demonstrate mutation-associated loss of enhancer signatures, characterized by concurrent loss of H3K4me1 and accessibility, with or without evident loss of H3K27ac, respectively. More molecular information on these specific clusters can be referred to (Yun et al., 2021) where the exemplar data were generated.

**Figure 1:**
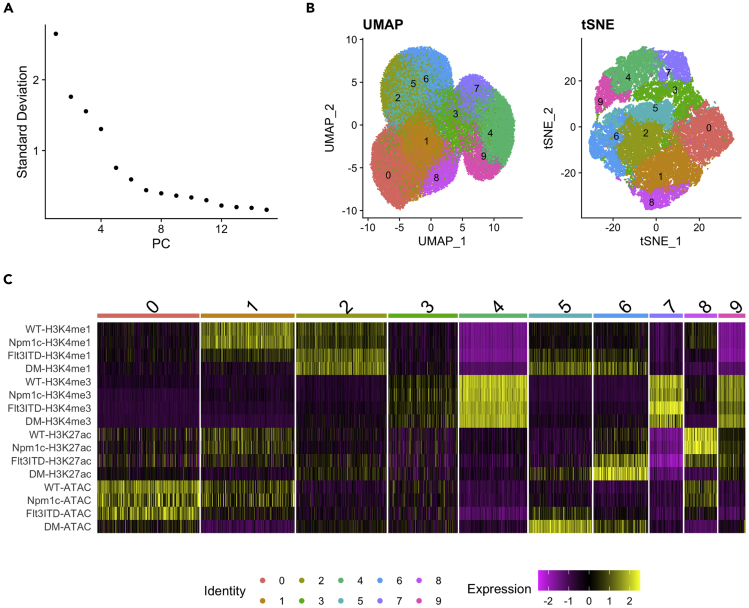
A multilayered approach to analyze dynamic chromatin marks upon mutational synergy induced leukemia (A) The elbow plot determines number of PCs to capture the variation in the data. (B) Non-linear dimensionality reduction by UMAP or tSNE clustering. (C) Heatmap shows individual clusters of CREs with dynamic patterns of chromatin modifications and accessibility across WT and mutant conditions.

### Data integration (ii)—Differential gene network

**Timing: 6 h**

In this step, we will link the CREs which demonstrate mutation-specific alteration of chromatin states to their associated genes with linear or spatial proximity. From step 6, Cluster-6 is selected as an exemplar group of CREs showing increased chromatin activity induced by mutations. The two mutations (*Npm1c* and *Flt3-ITD*) exert a strong synergy to induce a marked gain of H3K27ac, elevated levels of H3K4me1 and ATAC-seq, indicating the acquisition of enhancer signals by leukemia induction. Using the promoter-associated chromatin interaction data from pCHiC assays, Cluster-6 CREs are assigned to target genes when the CREs overlap with bait promoters or interaction fragments revealed by the pCHiC data. These target genes are then examined for differential expression analysis between mutant and WT samples, by checking them in the global analysis from step 2b. Since Cluster-6 CREs represent leukemia-specific gain of chromatin activity, the mutation-induced up-regulated genes linked to Cluster-6 CREs are further selected for gene ontology analysis, leading to the identification of leukemia-specific gene network related to chromatin alteration at 3D level.

7. Prepare the CRE annotation file using chromatin interaction information indicated by pCHiC data.
  a. Make a sample list (“makematrixsample.txt”) indicating the genotypes and the correspondent *.RDS* files (created by “runChicago.R” in step 3b) which contain promoter-associated DNA interactions generated by CHiCAGO, using the format below.

**Table:** 

[*GENOTYPE*]	[ *GENOTYPE_RDS_FILE*]

    ***Note:*** For each genotype, interaction data of two replicates are merged by running runChicago.R as input samples.
  b. Generate a consensus matrix of significant chromatin interactions (CHiCAGO score ≥5) detected in at least one genotype by running makePeakMatrix.R in CHiCAGO package (output file: “pCHiC_matrix.txt”).
    > Rscript makePeakMatrix.R --twopass ./makematrixsample.txt pCHiC_matrix > pCHiC_matrix.log
8. Identify specific target genes associated with Cluster-6 CREs which were identified in step 6 by utilizing chromatin interaction information.

> Rscript Cluster_CREs_genes_diffexp.R

# Annotation input files “Digest.mm10.rmap” and “pCHiC_fragID_Gene.txt” are provided in the KRT, while “pCHiC_matrix.txt” was generated in step 7b. Differential gene expression input file “WT.DM.PC.diffExp.csv” was generated in step 2b.

**Figure 2:**
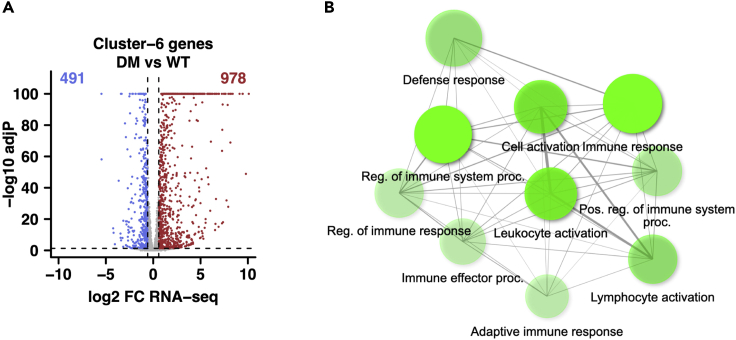
Altered expression of genes and gene network linked to DM-specific chromatin alteration (A) Differential expression of genes associated with Cluster-6 CREs showing leukemia-specific gain of chromatin activity. (B) Top 10 enriched network pathways of DM leukemia upregulated genes associated with Cluster-6 CREs.

***Note:*** The search species is set for “Mouse” and “GO Biological Process” is selected as target pathway database, with default parameters (FDR cut-off at 0.05) and setting 10 pathways to show. The output plot (Figure 2B) is generated under the tab Network.

***Note:*** Annotation is achieved by the exploration of pCHiC data (“pCHiC_matrix.txt” from step 7b), which include genomic coordinates of gene promoters (as “bait” fragment) and their interacting regions (as other end “oe” fragment). Next, by intersecting CREs with either “bait” or “oe” fragments, the target genes associated with specific CREs can be identified. These genes are further analyzed for altered expression by combined mutations (DM leukemia) in comparison to WT (Figure 2A, and the output file “Cluster-6_genes_DMvsWT_diffexp.txt”). Up- or down-regulation is defined as fold-change ≥1.5 and adjP <0.05. This step can be achieved by running custom scripts (“Cluster_CREs_genes_diffexp.R”).

9. Select the upregulated genes from previous step (the file “Cluster-6_DMvsWT_upgenes.txt” from step 8) to load into web server ShinyGO v0.76 (http://bioinformatics.sdstate.edu/go/) for gene network or pathway analysis.

## Expected outcomes

This protocol provides a novel and informative way to analyze multilayered chromatin profiling data from wildtype and mutant samples and to identify aberrant gene network and pathways induced by mutational synergy through 3D chromatin alteration. Using exemplar data, this approach allows the identification of multiple clusters of CREs (open chromatin regions) which are clearly separated from each other and indicate specific patterns of chromatin dynamics across WT and mutant conditions as shown in Figure 1. Furthermore, a representative group of CREs (Cluster-6) demonstrating leukemia-specific gain of chromatin activity are linked to their proximal genes at both linear and spatial levels. This facilitates the identification of genes which demonstrate leukemia-specific expression changes and suggest common pathways involved in DM leukemia as illustrated in Figure 2.

### Statistical analysis

Statistical analysis in the protocol is specified in detail in each step if relevant. Statistical calculations for differential gene expression analysis are performed with DESeq2, generating two-tailed and multiple testing corrected P (with the Benjamini and Hochberg method, adjP), with *adjP* ≤0.05 being considered statistically significant. Other statistical computation involves ATAC-seq peak calling (*p* value set for 1e-20 when using MACS2), identification of significant chromatin interaction profiled by pCHiC (CHiCAGO score ≥5).

## Limitations

The current protocol has been established and validated for the multiomics data analysis on active chromatin modifications (the marks for active CREs including enhancers and promoters such as H3K4m1, H3K4me3 and H3K27ac) at open chromatin regions. However, repressive chromatin marks (e.g., H3K9me2/3, H3K27me3) may be not a suitable source data for this approach without any optimization. Instead, consensus peak sets pooled from all profiled repressive chromatin marks can be computed to generate a compendium of repressive chromatin regions using a similar design. Therefore, though not yet tested, this multilayered analytical approach may serve other type of chromatin analysis.

## Troubleshooting

### Problem 1

Some software and algorithms used in this protocol were installed and tested on their old versions, leaving potential issues on the compatibility and reproducibility when running on the latest versions.

### Potential solution

We commented on this issue in the Note of the software section. In brief, all the R custom scripts run seamlessly with the latest R packages and latest R version (v.4.1.3), and the same results can be reproduced. And we anticipate the newer versions of other software and algorithms for NGS reads processing will retain the main analytical and statistical power with the same key parameters, though not yet been tested on our exemplar data. Nevertheless, we cannot confirm that any compatibility issues may not be encountered when running the latest versions of some software or algorithms.

### Problem 2

The peak calling for ATAC-seq data using MACS2 was rather simplified and may not reflect the actual size of nucleosome-free regions, although this might be acceptable for downstream integrative analysis.

### Potential solution

We commented on this issue in step 1c. As our exemplar ATAC-seq data are single-end reads and the fragment size was not determined, we chose to run MACS2 without modeling the fragment length by adding the parameter --nomodel, which, by default, extends all the reads for 200 bp. Ideally, this may be less problematic when processing paired-end ATAC-seq data.

### Problem 3

The making of the 2-kb bins at ATAC-seq consensus peak sets across all samples requires a complex processing which is possibly problematic if peak summit is not properly identified (step 4).

### Potential solution

The key to identify the consensus peaks summit requires the preparation of two lists: 1) a list indicating which sample has maximal ATAC-seq signal at each peak; 2) a list of all peak summits of individual samples. Overlapping these two lists will output the exact peak summit for each consensus peak.

### Problem 4

Annotation of specific CREs to target genes using chromatin interaction information requires serial steps to process pCHiC data. Improper preparation of relevant annotation files (step 8) may occur and cause problems to identify the right genes.

### Potential solution

First of all, instead of using the individual interaction profile of each sample, we use the consensus profiles to represent all interactions across all four cellular conditions. Second, we separately intersect the CREs with bait promoter fragments as well as other-end (oe) interaction fragments of pCHiC (being HindIII digestion fragments), and these overlapped oe fragment were further linked to their associated bait promoters using the interaction data.

### Problem 5

All the exemplar data sets include two biological replicates, to reduce the experiment bias and increase the statistical confidence of the analysis performed with this protocol. However, this protocol did not test data sets either with only a single run or with more replicates of experiment settings, leaving a potential issue on processing variable number of replicates.

### Potential solution

In the protocol we processed the biological replicates at different stages for different purpose. For example, for differential expression analysis, the replicates were treated separately for statistical calling, whereas for the construction of chromatin interaction matrix, replicates were merged to maximize the power of identifying chromatin interactions. Therefore, the way of dealing with replicate should be individually considered for each experiment stage and analysis purpose.

## Resource availability

### Lead contact

Further information and requests for resources and reagents should be directed to and will be fulfilled by the lead contact, Dr. Brian J. P. Huntly (bjph2@cam.ac.uk).

### Materials availability

This protocol does not require any newly generated materials associated with this protocol.

## Data Availability

This study did not generate any new data. All exemplar source data were published and were downloaded from Gene Expression Omnibus (GEO) database (GSE146616, GSE146662, GSE146663, GSE146668). All computation pipelines or custom code are available at https://github.com/haiyang-yun/3D_chromatin_in_AML (archived also on Zenodo: https://doi.org/10.5281/zenodo.7090655).
